# Fecal microbiota profiling in organic and conventional dairy farms differing in farm-level somatic cell counts and raw milk bacterial infections

**DOI:** 10.3389/fvets.2025.1734020

**Published:** 2026-01-12

**Authors:** Sung Jae Kim, Younghye Ro, Geun-Shik Lee, Keiichiro Kizaki, Atsushi Kimura, Yo-Han Kim

**Affiliations:** 1 Department of Companion Animal Health, Kyungbok University, Namyangju, Republic of Korea; 2 College of Veterinary Medicine and Institute of Veterinary Science, Kangwon National University, Chuncheon, Republic of Korea; 3 Cooperative Department of Veterinary Medicine, School of Veterinary Medicine, Iwate University, Morioka, Iwate, Japan

**Keywords:** fecal bacterial community, intramammary infection, organic and conventional farms, somatic cell count, subclinical mastitis

## Abstract

This study investigated the fecal bacterial communities in commercial dairy farms with varying milk quality, defined by differences in somatic cell counts, to elucidate their association with productive performance and the presence of intramammary bacterial infections. Four dairy farms, selected to represent diverse management environments, included one organic farm (OF) and three conventional farms (CF1, CF2, and CF3), with comparable parity ranges and days in milk, while somatic cell counts across farms ranged from 52.9 to 390.3 × 10^3^ cells/mL. Fecal bacterial community analysis identified 13 phyla and 190 genera, among which Order *Lachnospirales* (o_*Lachnospirales*), genus *UCG-01*0 (g_*UCG-010*), and genus *Rikenellaceae RC9 gut group* (g_*Rikenellaceae_RC9_gut_group*) showed the highest linear discriminant analysis (LDA) scores in OF, CF2, and CF3, respectively. Predicted functional pathway analysis of the fecal bacterial community identified 19 Kyoto Encyclopedia of Genes and Genomes (KEGG) pathways with significant differences. The pathway ‘various types of N-glycan biosynthesis’ (*ko00513*), which may be associated with immune activity in cows, was most upregulated in CF2, whereas ‘steroid biosynthesis’ (*ko00100*), related to fat metabolism in the rumen, was most downregulated in CF3 compared to OF. Correlation analyses of shared core taxa and milk parameters revealed that g_*Rikenellaceae_RC9_gut_group*, g_*UCG-010*, and g_*UCG-005*, which are closely linked to cellulose digestion and energy metabolism, showed significant correlations with milk urea nitrogen and milk protein content. Among the KEGG pathways, indole alkaloid biosynthesis (*ko00901*) and betalain biosynthesis (*ko00965*), both associated with gut health, were positively correlated with milk yield. Subclinical mastitis infection rates ranged from 27.8 to 58.1% per farm and 9.72 to 25.6% per quarter, with *Staphylococcus chromogenes* being the most prevalent bacterial isolate, followed by *S. aureus*, *S. simulans*, and *S. epidermidis*. Farm-level similarity and dissimilarity analyses revealed statistically significant differences in fecal bacterial community structures, predicted functions, and distributions of raw milk bacterial infections. These findings indicate that the distribution and functional potential of fecal bacterial communities are closely associated with milk quality parameters, whereas their composition and the distribution of intramammary pathogens are highly farm-specific, highlighting the need for further research to clarify their relationship.

## 1 Introduction

In dairy cattle, the gastrointestinal microbiota plays a crucial role in growth, health, productivity, and disease susceptibility throughout all stages of their productive life (1–3). In addition, the composition and structure of the fecal microbiota in dairy cows vary not only with the type and proportion of feed (4–6) but also with the overall nutritional composition of the diet (7, 8). In particular, the fecal microbiota, in association with productive performance, exhibits dynamic compositional and functional shifts during key physiological stages, including the transition, lactation, and dry periods (4, 5). Furthermore, because dairy cattle substantially depend on the gastrointestinal microbiota for efficient digestion, a thorough understanding of its functions is critically important (4). Notably, the fecal microbiota serves as an indicator of management-related factors, including both milk yield and milk quality (6–9).

Recent studies have primarily examined the role of the fecal microbiota in production performance, yet their potential contribution to the pathogenesis of bovine mastitis has also been proposed (8–10). It has been reported that inulin supplementation in dairy cows increases the production of propionate and butyrate while decreasing proinflammatory lipid oxidation products in feces. These changes suppress serum triglyceride and low-density lipoprotein concentrations and ultimately mitigate inflammatory responses by upregulating serum proteins related to immune response, lipid transport, and antioxidative stress, while downregulating serum acute-phase proteins in cows supplemented with inulin (11). Also, sialic acid–driven dysbiosis of the fecal microbiota can play a causative role in the development of mastitis in mouse model (12), and that subsequent lipopolysaccharide (LPS) translocation resulting from such dysbiosis may serve as a key mechanism of mastitis in cow (13). In addition, certain taxa may also participate in bacterial transmission through the gut-to-milk pathway (9), a hypothetical mechanism that has not been fully elucidated.

Alongside those findings, recent studies have shown that non-aureus *Staphylococci* (NAS), or coagulase-negative *Staphylococci* (CNS), have emerged as important causative agents of subclinical mastitis, frequently isolated from well-managed dairy herds (11, 14). The gastrointestinal tract appears to act as a reservoir and transmission route to the mammary glands (15, 16), and fecal microbiota transplantation models have linked gut microbiota to mastitis symptoms (17–19). In particular, several studies have reported the translocation of immune or bacterial components from the gut to the mammary gland via the entero-mammary pathway, highlighting its potential role in shaping the milk microbiota and influencing udder health (20–22). For example, distinctive gastrointestinal microbiota shaped by specific diets can affect the milk microbiome and consequently milk quality (20), and the secretion of intestinal bacterial components into milk may further support an endogenous entero-mammary pathogenic route in lactating cows (22). Furthermore, the heritability of rumen and fecal microbiota among Holstein cows (1, 23) suggests that microbial community composition may influence mastitis incidence. Consequently, the evaluation of intramammary infections such as subclinical mastitis is an important criterion for assessing milk quality, because NAS or CNS can contribute to elevated somatic cell counts in milk (24).

In conclusion, a substantial number of studies have suggested a potential link between the fecal microbiota and milk production and quality. However, the comprehensive association with variations in somatic cell count (SCC) and particularly intramammary infection (subclinical mastitis) status in commercial dairy farms remains poorly understood. Therefore, this study aims to profile the fecal microbiota in commercial dairy farms differing in farm-level somatic cell counts and raw milk bacterial infection status, and to elucidate its associations with various milk parameters.

## 2 Materials and methods

The Institutional Animal Care and Use Committee of Kangwon National University Laboratory (KW-231106-1; Chuncheon, Korea) approved the use of animals and all experimental protocols.

### 2.1 Sampling and measurements

Four commercial dairy farms, including one organic farm (OF) and three conventional farms (CF1, CF2, and CF3), were used in this study. The organic farm was certified as an organic dairy farm in December 2021 and operated in accordance with the certification guidelines of the organic dairy farm (National Agriculture Products Quality Management Service) a year before certification.

The farms used in this study were selected based on the monthly milk test results obtained from the Dairy Cattle Improvement Center^1^ in South Korea. Production information regarding day in milk (DIM), milk yield, SCC, composition of milk fat, protein, total milk solids, and milk urea nitrogen (MUN) concentrations was also obtained from the center’s monthly test results (Table 1). In this study, 119 Holstein cows were included, and skilled veterinarians aseptically collected quarter milk samples (n = 476) from 43 (a total of 45), 18 (of 18), 25 (of 55), and 31 (of 58) cows in the OF, CF1, CF2, and CF3 farms, respectively. No clinical symptoms were noted in any of the cows in this study.

**Table 1:** Parity, day in milk, milk yield, and milk compositions in Holstein cows in the organic dairy (OF) and conventional dairy farms (CF1, CF2, and CF3).

Items	Groups^1^				SEM	*p*-value
	OF (*n* = 45)	CF1 (*n* = 18)	CF2 (*n* = 55)	CF3 (*n* = 58)		
Parity	1.98	2.06	2.14	2.39	0.19	0.710
DIM^2^ (days)	216.9	196.6	224.4	224.4	21.4	0.886
Milk Yield (kg/cow)	26.4	28.7	31.8^a^	33.5^a^	1.38	0.001
SCC^3^ (×10^3^)	307.8	52.9^a^	125.4	390.3	53.2	<0.001
Milk Fat (%)	3.86	4.09	4.48^a^	3.82	0.12	<0.001
Milk Protein (%)	3.45	3.41	3.59	3.44	0.06	0.036
Total Milk Solids (%)	8.89	8.70	8.68	8.97	0.06	0.004
MUN^4^ (mg/dL)	14.9	12.3^a^	15.4	11.3^a^	0.34	<0.001

^1^OF, Organic Farm; CF, Conventional Farm; ^2^DIM, Day In Milk; ^3^SCC, Somatic Cell Count; ^4^MUN, Milk Urea Nitrogen. ^5^Kruskal-Wallis test, followed by Dunn’s multiple comparison method, was used to determine the effect of Farm. ^a^Denotes significant difference (*p* < 0.05) compared with OF.

Ten microliters of the quarter samples were inoculated onto blood agar plate (BAP), and all plates were aerobically incubated for 24 h at 37 °C as previously described (25). Subsequently, one pure colony of culture-positive plates was aerobically sub-cultured for 20 to 24 h at 37 °C on BAP for species identification using matrix-assisted laser desorption/ionization time-of-flight (MALDI-TOF) mass spectrometry (MS) assay (VITEK® MS PRIME; BIOMERIEUX, France). One pure colony was selected and added to 1 μL of the matrix solution, dried, and assessed using MALDI-TOF MS assay. The spectral data were subsequently analyzed by comparison with the typical spectra.

### 2.2 16S rRNA sequencing of the fecal bacterial community

A total of 41 rectal fecal samples were collected from dairy cows in the OF (*n* = 10), CF1 (*n* = 10), CF2 (*n* = 11), and CF3 (*n* = 10) farms, from which milk samples had been collected to elucidate the relationship between fecal microbiota and milk quality parameters. Genomic DNA was extracted from the fecal samples using the QIAamp DNA Mini Kit (Qiagen, Hilden, Germany) following the manufacturer’s instructions with minor modifications (20). Approximately 469 bp encompassing the V3 and V4 hypervariable regions within the 16S rRNA gene was subsequently amplified using two universal primers with adapter overhang sequences: V3-F, ′5-TCGTCGGCAGCGTCAGATGTGTATAAGAGACAG CCTACG GGNGGCWGCAG-3′, and V4-R, ′5-GTCTCGTGGGCTCGGAGATGTGTATAAGAGACAGGACTACHVGGGTA TCTAATCC-3′. 95 °C for 3 min, then 25 cycles of 95 °C for 30 s, 55 °C for 30 s, and 72 °C for 30 s, with a final extension of 72 °C for 5 min, were the thermal cycling conditions. The 16S rRNA sequencing was conducted using an Illumina MiSeq platform (San Diego, CA, USA). Raw Illumina MiSeq data were classified using an index sequence and a paired-end FASTQ file was created for each sample. The obtained sequencing data were deposited in the Sequence Read Archive of the National Center for Biotechnology Information and can be accessed via the SRA BioSample accession number SAMN47283394^2^.

### 2.3 Sequencing data processing

The sequence data were processed using QIIME2 (v. 2024.5). The paired-end sequences were aligned into a single sequence following adaptor removal using Cutadapt (v. 3.4), and chimeric sequences were eliminated using the DADA2 consensus technique to extract amplicon sequence variants (ASVs). Subsequently, the taxonomic assignment was conducted with the SILVA silva138 AB V3–V4 classifier using the feature-classifier classification-sklearn plugin. Using the default pipeline of PICRUSt2, predicted functional pathways were inferred based on representative sequences and tabulated raw count data from the 16S rRNA gene data. Functional differences between the groups were collectively examined based on the Kyoto Encyclopedia of Genes and Genomes pathway (KEGG).

### 2.4 Statistical analyses

The Kruskal–Wallis test (nonparametric statistical test), followed by Dunn’s multiple comparison method, was used to evaluate production information, bacterial taxa, and the effect of the farm. Linear discrimination (LDA) and effect size (LEfSe) analyses were conducted to identify markers that varied in abundance between the groups using the biomeMarker package in R studio (v 4.4.1). The LEfSe analysis was set as normalization = counts per million mapped reads, Kruskal–Wallis test cut-off = 0.01, Wilcoxon test cut-off = 0.01, LDA score cut-off = 4. After flooring the fractional counts to the nearest integer, the raw KEGG pathway output from PICRUSTt2 (21) was analyzed using DESeq2 (22) with default parameters to determine whether the predicted functional pathway of the fecal bacterial community varied between OF and other farms. The inferred probable functional pathways were determined to be differentially abundant at a false discovery rate (FDR)-rate-corrected significance level of < 0.05. Non-metric multidimensional scaling (NMDS) plots were generated for the bacterial composition and KEGG pathways assigned by PICRUSt2 using the vegan package in R studio (v 4.4.1). Principal component analysis (PCA) plots were constructed for the raw milk bacterial infection rates using the R package ggbiplot (v 4.4.1), including the raw milk bacterial infection distributions for each farm. Dissimilarities in the fecal bacterial communities and predicted functions were examined using the ANOSIM test in QIIME2, and similarities in the distributions of raw milk bacteria were examined using Spearman’s correlation test in Prism. Statistical significance was set at *p* < 0.05.

## 3 Results

### 3.1 Farm-level milk production information

A total of 4 dairy farms were selected based on the similar parity range (1.98 to 2.39 parities; *p* = 0.710) and DIM (196.6 to 224.4 days; *p* = 0.886) but different SCC (52.9 to 390.3 × 10^3^; *p* < 0.001) in the farm scale (Table 1). Significant differences were also observed in milk fat (3.82–4.48%; *p* < 0.01), milk protein (3.41 to 3.59%; *p* = 0.036), total milk solid (8.68–8.89%; *p* < 0.01), and MUN (11.3–15.4 mg/dL; *p* < 0.01) among the farms (Table 1).

### 3.2 Fecal bacterial community composition and diversity

**Figure 1:**
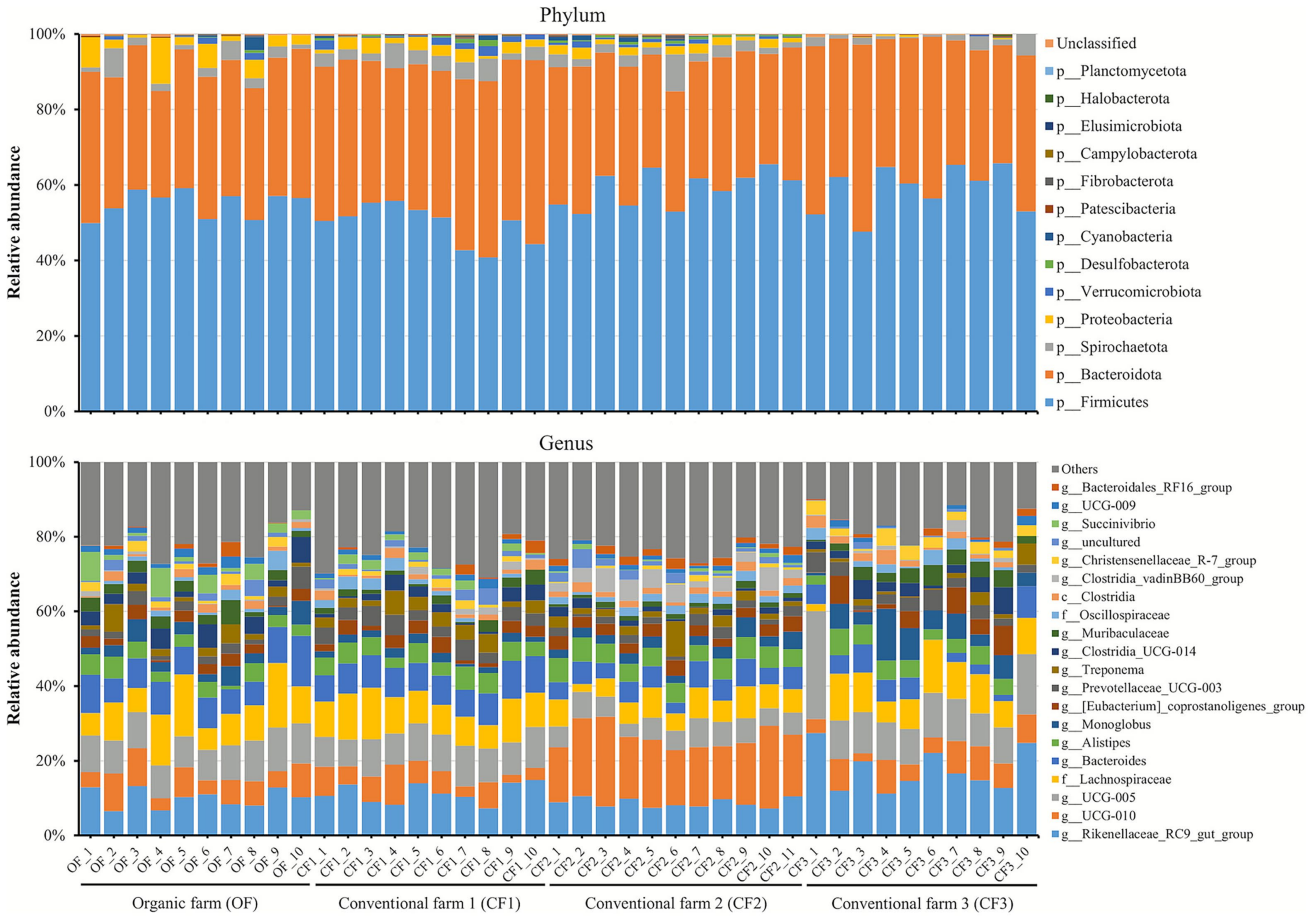
Relative abundances of bacterial phyla and genus profiles in Holstein cows in the organic dairy (OF) and conventional farms (CF1, CF2, and CF3). Data are indicated as percentages of the total identified sequences per individual cow.

Figure. 1 summarizes the relative abundances of the fecal bacterial phyla and genera in individual cows. At the phylum level, the most abundant were p_*Firmicutes*, followed by p_*Bacteroidota*, accounting for 91.2 to 97.5%. In the genus level, the most abundant were *f_Lachnospiraceae* in the OF (10.4%) and CF1 (9.73%), g_*UCG-010* in the CF2 (17.7%), and g_*Rikenellaceae_RC9_gut_group* in the CF3 (17.6%) (Table 2). All bacterial phyla (5 of 5) and 15 of 20 bacterial genera with proportions > 1% demonstrated significant differences among the dairy farms (*p* < 0.05). The relative abundance of g_*UCG-010* was significantly higher than that of g_*UCG-005*, *f_Lachnospiraceae*, *g_Bacteroides*, g_*Clostridia_UCG-014*, g_*Muribaculaceae*, g_*Clostridia_vadinBB60_group*, and g_*Succinivibrio* in CF2 compared to OF (*p* < 0.05). The relative abundance of the g_*Rikenellaceae_RC9_gut_group* was significantly higher and those of p_*Proteobacteria*, g_*Rikenellaceae_RC9_gut_group*, g_*Bacteroides*, and g_*Succinivibrio* were significantly lower in CF3 than in OF (*p* < 0.05).

**Table 2:** Distribution of raw milk bacterial infections in Holstein cows in the organic dairy (OF) and conventional dairy farms (CF1, CF2, and CF3).

Items	Bacterial infection rates^1^			
	OF (*n* = 180)	CF1 (*n* = 72)	CF2 (*n* = 100)	CF3 (*n* = 124)
Species^2^				
Coagulase negative *Staphylococci*	23 (12.8%)	6 (9.72%)	13 (13.0%)	24 (19.4%)
*Staphylococcus chromogenes*	10 (5.56%)	3 (4.17%)	8 (8.00%)	13 (10.5%)
*Staphylococcus simulans*	5 (2.78%)	0 (0.00%)	1 (1.00%)	5 (4.03%)
*Staphylococcus epidermidis*	8 (4.44%)	2 (2.78%)	0 (0.00%)	0 (0.00%)
*Staphylococcus haemolyticus*	0 (0.00%)	0 (0.00%)	2 (2.00%)	4 (3.23%)
*Staphylococcus muscae*	0 (0.00%)	0 (0.00%)	0 (0.00%)	2 (1.61%)
*Staphylococcus xylosus*	0 (0.00%)	0 (0.00%)	1 (1.00%)	0 (0.00%)
*Staphylococcus hyicus*	0 (0.00%)	1 (1.39%)	1 (1.00%)	0 (0.00%)
*Staphylococcus aureus*	18 (10.0%)	0 (0.00%)	6 (6.00%)	0 (0.00%)
*Enterococcus faecalis*	1 (0.56%)	0 (0.00%)	2 (2.00%)	3 (2.42%)
*Streptococcus dysgalactiae*	0 (0.00%)	0 (0.00%)	1 (1.00%)	1 (0.81%)
*Aerococcus viridans*	0 (0.00%)	0 (0.00%)	0 (0.00%)	1 (0.81%)
*Streptococcus pluranimalium*	1 (0.56%)	0 (0.00%)	0 (0.00%)	0 (0.00%)
*Enterococcus saccharolyticus*	1 (0.56%)	0 (0.00%)	0 (0.00%)	0 (0.00%)
*Streptococcus uberis*	0 (0.00%)	0 (0.00%)	0 (0.00%)	1 (0.81%)
Unidentified^4^	0 (0.00%)	1 (1.39%)	0 (0.00%)	1 (0.81%)
Total	44 (24.4%)	7 (9.72%)	22 (22.0%)	31 (25%)

^1^OF, Organic Farm; CF, Conventional Farm. ^2^Species identification was obtained using MALDI-TOF MS on a Vitek MS platform. ^3^A total of 180, 72, 100, and 124 raw milk samples were collected from OF (45 cows), CF1 (18 cows), CF2 (25 cows), and CF3 (31 cows), respectively. ^4^Raw milk bacterial culture positive in blood agar plate, but unidentified by MALDI-TOF MS assay.

### 3.3 LEfSe analysis for significant bacterial markers in each farm

**Figure 2:**
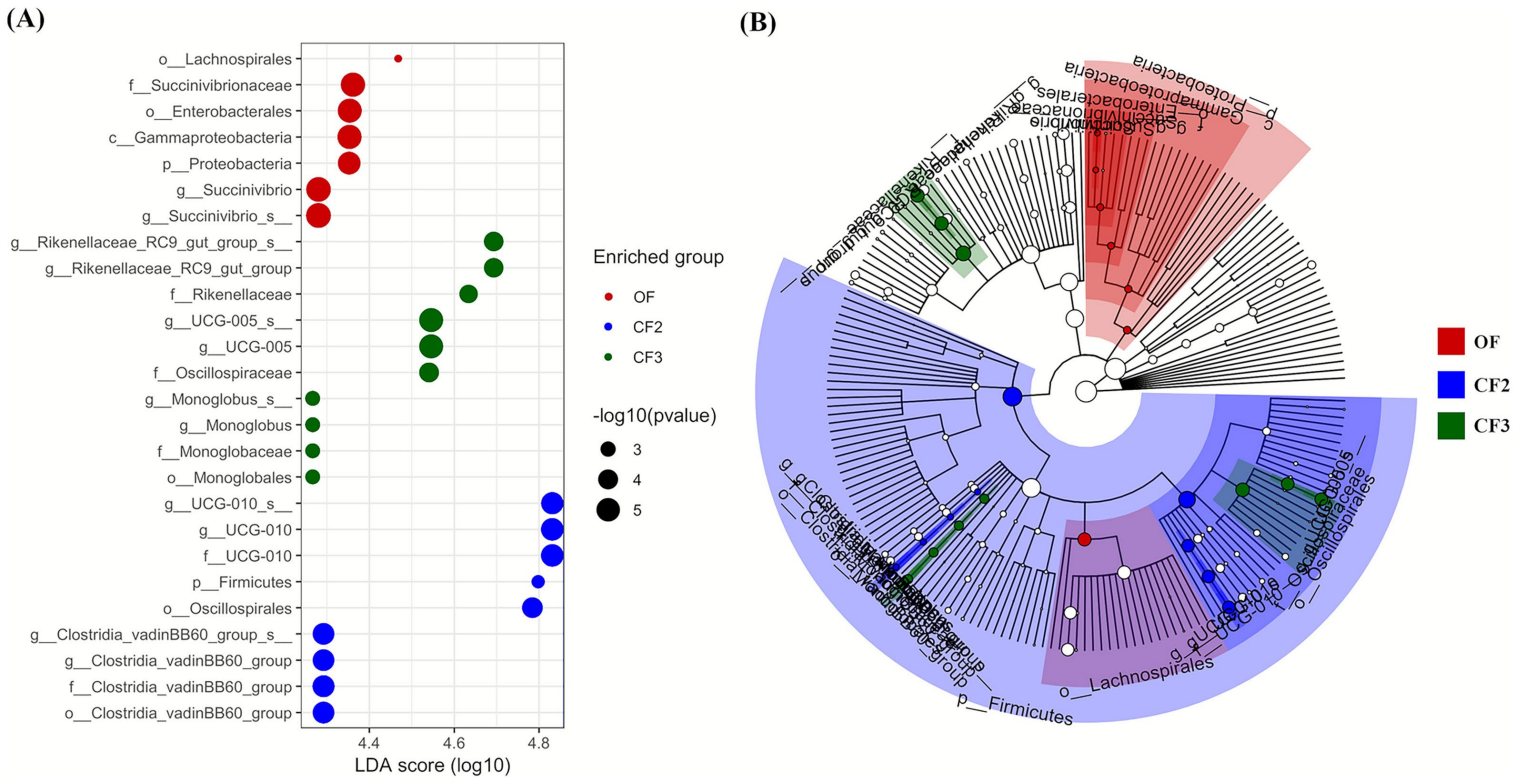
Linear discriminant analysis (LDA) effect size (LEfSe) analysis conducted on Holstein cows in the organic dairy (OF) and conventional farms (CF1, CF2, and CF3). The abundance plot on count reads normalized by LDA score dot plot **(A)** and cladogram **(B)** were visualized based on the significant markers (*p* < 0.01) and LDA score (>4).

In the LEfSE analysis, several markers were identified in OF, CF2, and CF3 (*p* < 0.05), whereas no markers were observed in CF1 (Figure. 2). g_*UCG-010_*s_, g_*UCG-010*, and f_*UCG-010* in CF2 demonstrated the highest LDA scores among all the markers. g_*Rikenellaceae_RC9_gut_group_*s and g_*Rikenellaceae_RC9_gut_group* revealed the highest LDA scores in CF3, whereas o_*Lachnospirales* showed the highest LDA scores in OF.

### 3.4 Predicted functional pathway analysis of fecal bacterial community

Raw KEGG pathway abundance was determined using PICRUSt2. Metabolism (67.2–69.6%) was the most abundant pathway at level 3, followed by Genetic Information Processing (17.4–18.5%), Environmental Information Processing (6.84–8.73%), Cellular Processes (3.32–4.11%), Organismal Systems (1.21–1.37%), and Human Diseases (0.89–1.10%). Among the 210 KEGG pathways, 30 (FDR adjusted *p* value < 0.05, absolute value of fold-change > 2) were identified (Table 3). Compared to OF, the most upregulated KEGG pathway in CF1 and CF2 was “*ko00513*” (various types of *N*-glycan biosynthesis; FC = 4.54 and 4.93, respectively), while the most downregulated pathway was “*ko00100*” (Steroid biosynthesis; FC = −39.4) in CF3.

**Table 3:** Relative abundances of major bacterial phyla and genera (> 1% of total sequences) in Holstein cows in organic dairy (OF) and conventional farms (CF1, CF2, and CF3).

Items	Groups^1^				SEM	*P*-value^2^
	OF (*n* = 10)	CF1 (*n* = 10)	CF2 (*n* = 11)	CF3 (*n* = 10)		
Phylum						
p_*Firmicutes*	55.1	49.7	59.1	58.8	1.54	0.002
p_*Bacteroidota*	36.3	41.6	33.8	38.7	1.30	0.003
p_*Spirochaetota*	2.79	3.78	3.02	1.91	0.60	0.029
p_*Proteobacteria*	4.36	2.59	1.99	0.18^a^	0.44	<0.001
p_*Verrucomicrobiota*	0.45	1.24	0.66	0.06	0.18	0.001
Genus						
g_*Rikenellaceae_RC9_gut_group*	10.0	11.3	8.71	17.6^a^	0.95	<0.001
g_*UCG-010*	6.58	5.75	17.7^a^	6.41	0.87	<0.001
g_*UCG-005*	9.58	9.25	5.80^a^	12.7	0.76	<0.001
f_*Lachnospiraceae*	10.4	9.73	6.20^a^	9.02	0.93	0.012
g_*Bacteroides*	8.51	8.24	5.39^a^	4.43^a^	0.57	<0.001
g_*Alistipes*	3.80	4.72	5.36	4.06	0.41	0.051
g_*Monoglobus*	3.54	1.95	3.03	5.81	0.55	0.001
g_*[Eubacterium]_coprostanoligenes_ group*	2.41	2.64	3.18	3.30	0.50	0.778
g_*Prevotellaceae_UCG-003*	2.51	3.93	1.98	3.23	0.38	0.004
g_*Treponema*	2.65	3.50	2.78	1.69	0.59	0.021
g_*Clostridia_UCG-014*	3.58	2.13	1.26^a^	2.94	0.53	0.041
g_*Muribaculaceae*	3.05	1.90	1.32^a^	3.30	0.35	0.002
f_*Oscillospiraceae*	1.45	1.63	1.91	1.60	0.37	0.473
c_*Clostridia*	1.47	1.49	1.69	1.73	0.26	0.791
g_*Clostridia_vadinBB60_group*	0.38	0.88	4.03^a^	0.68	0.31	<0.001
g_*Christensenellaceae_R-7_group*	1.54	1.10	0.56	2.79	0.29	0.001
g_*uncultured*	1.48	1.70	1.91	0.45	0.34	0.003
g_*Succinivibrio*	3.35	1.54	0.52^a^	0.01^a^	0.32	<0.001
g_*UCG-009*	1.51	1.15	1.24	0.75	0.23	0.158
g_*Bacteroidales_RF16_group*	0.75	0.86	1.71	0.76	0.32	0.036

^1^OF, Organic Farm; CF, Conventional Farm. ^2^Kruskal-Wallis test, followed by Dunn’s multiple comparison method, was used to determine the effect of Farm. ^a^Denotes significant difference (*p* < 0.05) compared with OF.

### 3.5 Distribution of intramammary bacterial infections

The infection rates of subclinical mastitis in the cows and quarters were 58.1% (25/43) and 25.6% (44/172) in OF; 27.8% (5/18) and 9.72% (7/72) in CF1; 48.0% (12/25) and 22.0% (22/100) in CF2; and 58.1% (18/31) and 25.0% (31/124) in CF3, respectively (Table 2). The most prominent isolate in the OF was *S. aureus* (40.9%), followed by *S. chromogenes* (22.7%) and *S. epidermidis* (18.2%). In CF1, *S. chromogenes* was the most prominent isolate (42.9%), followed *by S. epidermidis* (28.6%) and *S. hyicus* (14.3%). In CF2, *S. chromogenes* was the most prominent (36.4%), followed by *S. aureus* (27.3%) and *Enterococcus faecalis* (9.1%). Similarly, in CF3, *S. chromogenes* was the most prominent (41.9%), followed by *S. simulans* (16.1%) and *E. faecalis* (12.9%). The prominence of *S. aureus* was not verified (Table 4).

**Table 4:** Predicted functional pathways (|fold change| > 2) from the PICRUSt2 analysis in comparison of the organic (OF) and conventional (CF1, CF2, and CF3) dairy farms.

KEGG pathway	Fold change^1^			Adjusted *P*-value^2^		
	CF1 *vs*. OF^3^	CF2 *vs*. OF	CF3 *vs*. OF	CF1 *vs*. OF	CF2 *vs*. OF	CF3 *vs*. OF
*ko00100*	−1.40	−2.86	−39.39	0.422	0.001	<0.001
*ko00513*	4.54	4.93	−1.61	0.037	0.006	0.558
*ko00624*	−2.92	2.79	1.12	0.246	0.113	0.907
*ko00791*	−4.03	−3.03	1.53	0.074	0.082	0.609
*ko00901*	−1.38	−4.91	1.36	0.707	0.006	0.712
*ko00941*	1.44	2.03	−2.98	0.707	0.313	0.151
*ko00965*	−1.38	−4.91	1.36	0.707	0.006	0.712
*ko03022*	1.18	−3.03	1.08	0.849	0.057	0.907
*ko03050*	1.18	−3.03	1.08	0.849	0.057	0.907
*ko04020*	3.02	−3.03	1.09	0.099	0.081	0.907
*ko04110*	1.17	−3.03	3.62	0.849	0.093	0.039
*ko04145*	1.17	−3.03	3.62	0.849	0.093	0.039
*ko04150*	1.17	−3.03	3.62	0.849	0.093	0.039
*ko04260*	−1.41	2.73	−2.62	0.681	0.086	0.125
*ko04510*	1.18	−2.58	1.08	0.849	0.091	0.907
*ko04512*	1.18	−2.40	1.08	0.849	0.112	0.907
*ko04514*	1.18	−2.58	1.08	0.849	0.091	0.907
*ko04614*	−1.10	−1.82	−2.58	0.916	0.359	0.212
*ko04622*	−3.27	−1.45	1.43	0.033	0.441	0.525
*ko04640*	1.18	−2.78	1.08	0.849	0.077	0.907
*ko04810*	1.18	−2.58	1.08	0.849	0.091	0.907
*ko04962*	1.18	−3.03	1.08	0.849	0.057	0.907
*ko04976*	1.17	−3.03	3.62	0.849	0.093	0.039
*ko05110*	−2.68	−1.34	−1.60	0.236	0.684	0.582
*ko05130*	1.17	−1.11	2.26	0.849	0.882	0.230
*ko05131*	1.17	1.05	4.92	0.868	0.947	0.026
*ko05160*	1.17	−3.03	3.62	0.849	0.093	0.039
*ko05410*	1.18	−2.58	1.08	0.849	0.091	0.907
*ko05412*	1.18	−2.58	1.08	0.849	0.091	0.907
*ko05414*	1.18	−2.58	1.08	0.849	0.091	0.907

^1^Fold change (FC) was calculated by comparison of OF with CF1, CF2, and CF3 (FC > 2.0). ^2^Adjusted *P*-value was calculated by false discovery rate method. ^3^OF, Organic Farm; CF, Conventional Farm.

### 3.6 Similarity and dissimilarity analyses of bacterial distributions

**Figure 3:**
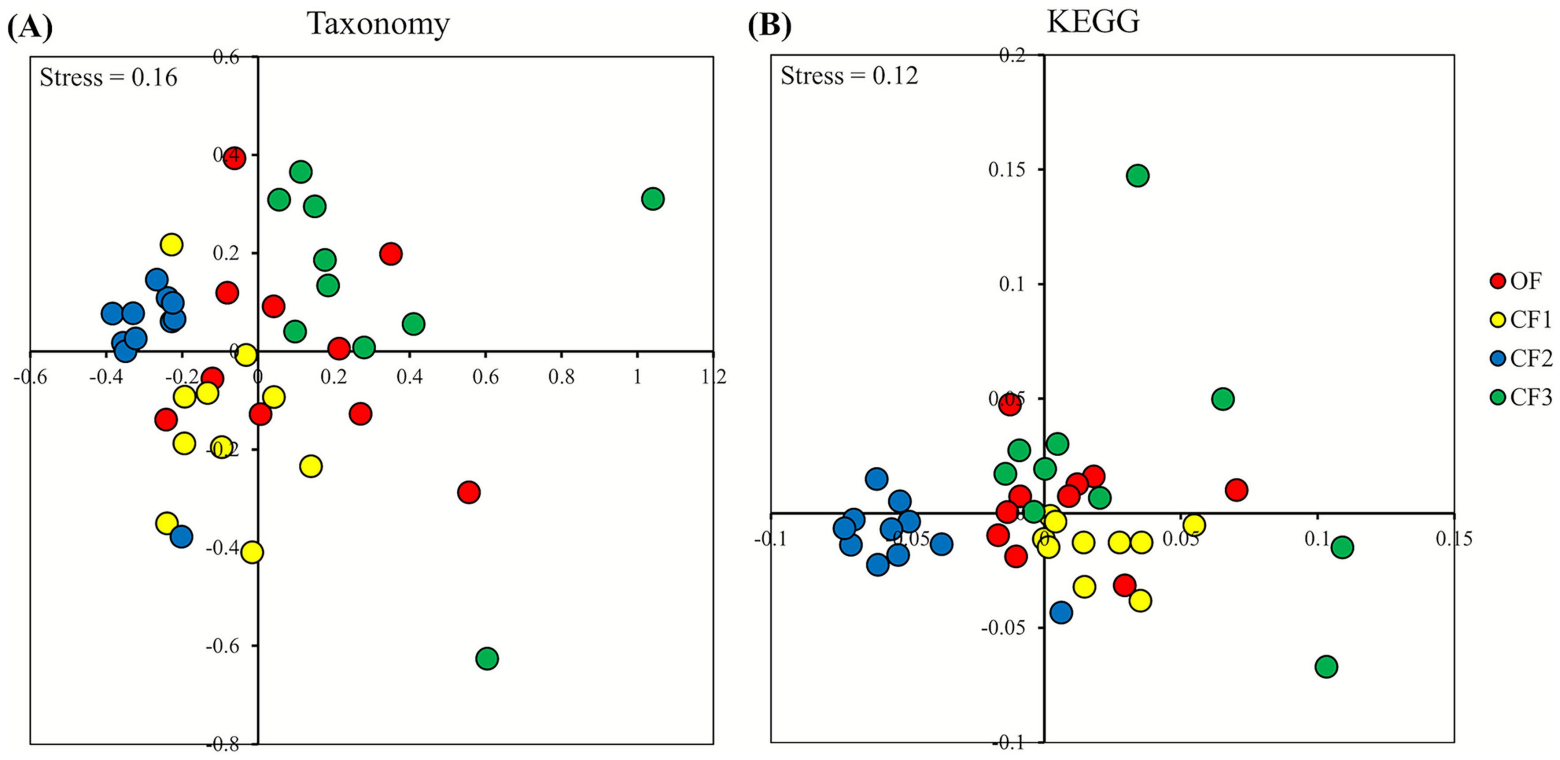
Non-metric multidimensional scaling (NMDS) plots for Holstein cows in the organic dairy (OF) and conventional farms (CF1, CF2, and CF3). NMDS plots were generated for **(A)** bacterial composition and **(B)** Kyoto Encyclopedia of Genes and Genomes (KEGG) pathways assigned by PICRUSt2. Stress values were 0.16 and 0.12 for bacterial taxonomy- and KEGG pathway-based ordinations, respectively.

The NMDS and PCA plots revealed similarities and dissimilarities in bacterial composition and KEGG pathways (Figure 3). In comparing groups, dissimilarity-based ANOSIM analysis in QIIME2 demonstrated that the fecal bacterial communities (OF *vs*. CF1, *R* = 0.28; OF *vs*. CF2, *R* = 0.79; OF *vs*. CF3, *R* = 0.73; CF1 *vs*. CF2, *R* = 0.79; CF1 *vs*. CF3, *R* = 0.73; CF2 *vs*. CF3, *R* = 0.77) and predicted functions of bacterial communities (OF *vs*. CF1, *R* = 0.21; OF *vs*. CF2, *R* = 0.41; OF *vs*. CF3, *R* = 0.17; CF1 *vs*. CF2, *R* = 0.67; CF1 *vs*. CF3, *R* = 0.36; CF2 *vs*. CF3, *R* = 0.33) are significantly (*p* < 0.01) separated in each other farms. Additionally, the similarity-based Spearman’s correlation test revealed that the distributions of raw milk bacterial infections were significantly (*p* < 0.01) different (OF *vs*. CF1, *R* = 0.36; OF *vs*. CF2, *R* = 0.34; OF *vs*. CF3, *R* = 0.01; CF1 *vs*. CF2, *R* = 0.19; CF1 *vs*. CF3, *R* = 0.00; CF2 *vs*. CF3, *R* = 0.41).

In the distributions of raw milk bacterial infections, PCA plots revealed that dairy farms were most influenced by *S. aureus*, *S. xylosus*, and *S. muscae* in the OF; *S. hyicus* and *S. epidermidis* in the CF1; *Aerococcus viridans* in the CF2; and *S. haemolyticus*, *Streptococcus pluranimalium*, *Enterococcus saccharolyticus*, *Streptococcus uberis*, *E. faecalis*, and *Streptococcus dysgalactiae* in CF3 (principal components 1 + 2, explaining 79.3% of the variance; Supplementary Figure S1).

### 3.7 Correlation analyses of the core bacterial ASVs and milk parameters

**Figure 4:**
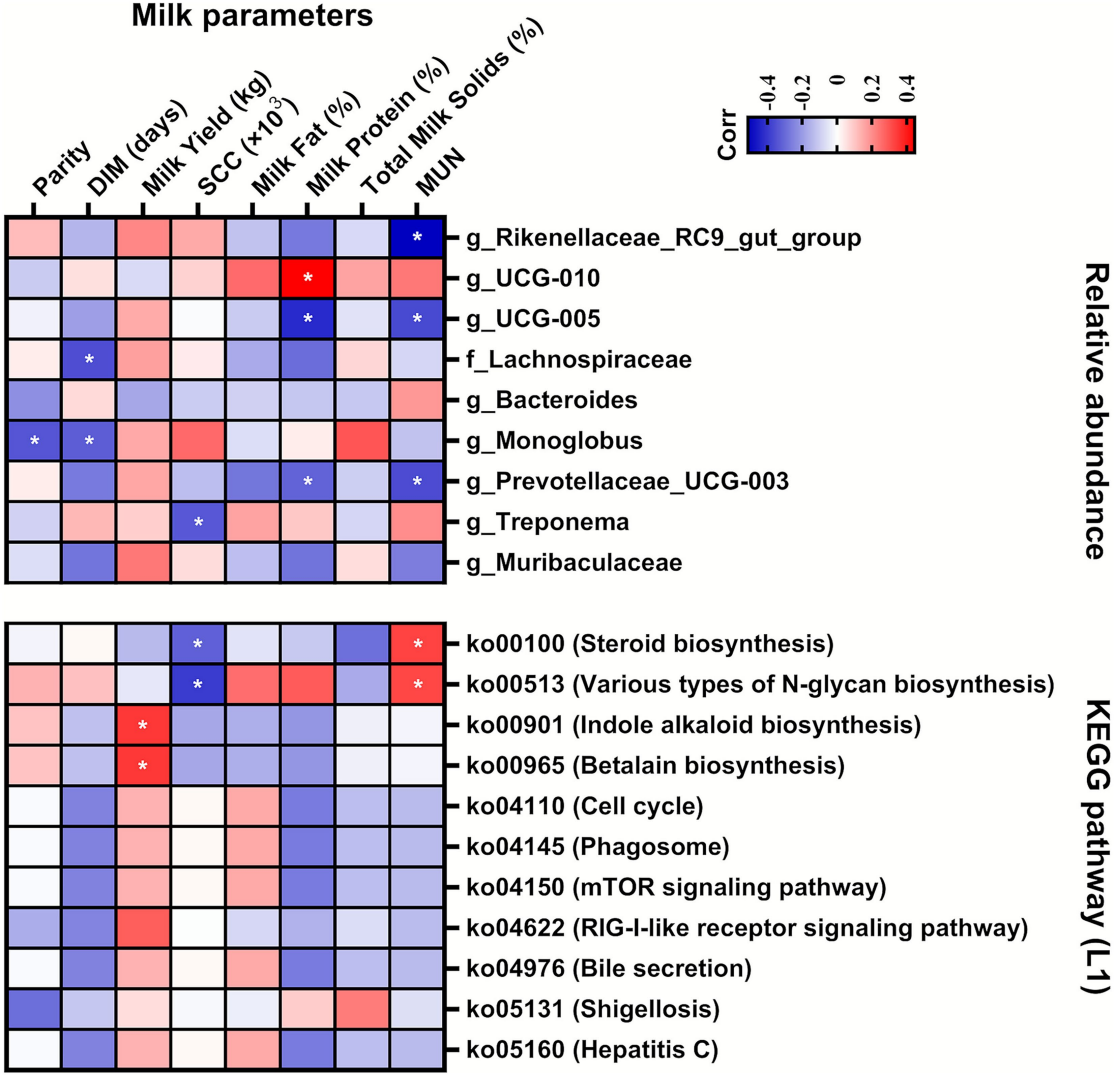
Correlation analyses between the production parameters and relative abundances of core amplicon sequence variants (ASVs) (shared by all samples) or significantly (*p* < 0.05) identified by Kyoto Encyclopedia of Genes and Genomes (KEGG) pathways. Cells are colored based on Spearman’s correlation analyses. Blue and red represent negative and positive correlations, respectively. *Significant correlation at *p* < 0.05. DIM: days in milk; SCC: somatic cell count; MUN: milk urea nitrogen; L`: level 1.

A total of 9 ASVs, shared by 100% of the samples, were identified regardless of cow or farm, such as g*_Rikenellaceae_RC9_gut_group*, g*_UCG-010*, *g_UCG-005*, f_*Lachnospiraceae*, g_*Bacteroides*, g_*Monoglobus*, g_*Prevotellaceae_UCG-003*, g_*Treponema*, and g_*Muribaculaceae*. Among the ASVs that were correlated (*p* < 0.05) with milk parameters, the relative abundance of g_*UCG-010* was positively (*r* = 0.44) correlated with milk protein, and negative correlations were identified in the relative abundances of g_*Rikenellaceae_RC9_gut_group* (with MUN, *r* = −0.51), g_*UCG-005* (milk protein, *r* = −0.43 and MUN, *r* = −0.37), f_*Lachnospiraceae* (DIM, *r* = −0.36), g_*Monoglobus* (Parity, *r* = −0.35 and MUN, *r* = −0.33), g_*Prevotellaceae_UCG-003* (milk protein, *r* = −0.32 and MUN, *r* = −0.36), and g_*Treponema* (SCC, *r* = −0.34) (Figure 4). Among the significantly (*p* < 0.05) identified 11 KEGG pathways among the farms, “*ko00100*” and “*ko00513*” were positively and negatively correlated with MUN (*r* = 0.33 and *r* = 0.32, respectively) and SCC (*r* = −0.32 and *r* = −0.40, respectively). “*ko00901*” (Indol alkaloid biosynthesis) and “*ko00965*” (Betalain biosynthesis) were positively correlated with the milk yield (*r* = 0.35 and *r* = 0.35, respectively) (Figure 4).

## 4 Discussion

We aimed to identify the correlation between the fecal bacterial community and milk quality in four dairy farms with varying somatic cell counts (52.9–390.3 × 10^3^ cells/mL) and intramammary bacterial infections, including *S. aureus* and seven CNS isolates, three *Streptococcus* isolates, two *Enterococcus* isolates, and one *Aerococcus* isolate. Although milk composition differed possibly due to detailed management strategies across farms in this study, these variations fell within the range reported in previous studies conducted in Korea (26, 27) and other countries (28, 29). Moreover, parity and DIM did not differ significantly among the farms in the present study. Therefore, the farms selected in this study, including both organic and conventional farms, are representative of the typical management practices commonly observed in Korean dairy farms. Furthermore, the bacterial composition of each farm was analyzed using the LefSe method, while the predicted functional profiles of the bacterial communities were compared between the organic and conventional farms.

In the fecal bacterial community, the predominant taxa belonged to the *Firmicutes* and *Bacteroidota* phyla. In addition, the major families including g_*Rikenellaceae_RC9_gut_group*, g_*UCG-010*, g_*UCG-005*, and *Lachnospiraceae*, all of which were further identified as key biomarkers through LEfSE analysis for each farm. The *Rikenellaceae_RC9_gut_group* identified in this study has been consistently reported in the literatures as one of the dominant bacterial taxa accounting for more than 8% of fecal microbiota in dairy cows (30, 31). Moreover, previous studies have shown that the *Rikenellaceae_RC9_gut_group* is associated with cellulase activity in the rumen (31), can utilize crude fiber as a carbohydrate source to produce acetate and propionate (32), and is implicated in energy metabolism and inflammation in cattle feces (30). Both g_*UCG-010* and g_*UCG-005*, members of the family *Ruminococcaceae* under the phylum *Firmicutes*, dominated the fecal bacterial community in this study, consistent with previous reports (30, 33). Furthermore, these taxa have been associated with energy metabolism and inflammation in cattle feces (30), and with fatty acid metabolism during yeast supplementation (33). Meanwhile, the *Lachnospiraceae* family is involved in butyrate production (34) and contribute to intestinal mucosal protection, inflammation regulation, and energy metabolism (6), consequently helping to maintain microbial diversity and stability in the gut and thereby enhancing feed efficiency, health, and productivity in dairy cows (35). Therefore, although the predominant taxa identified may vary according to herd nutritional management, the fecal bacterial community structure in the present study aligns with findings reported in various previous studies (6, 30–35), and thus suggests its important role in maintaining gastrointestinal health, with no evidence of dysbiosis observed.

Although only a few studies were reported regarding functional analysis result on fecal bacterial community in dairy cow, our findings showed that the most prevalent KEGG pathways were metabolism (level 1) and glycolysis/gluconeogenesis (level 3), which is consistent with that found in previous studies (4, 36). Specifically, the most upregulated various types of N-glycan biosynthesis in the CF1 and CF2 is associated with gut microbiota-derived glycans that might have direct effect on host immune activity in human (37, 38), and also related to the immunoglobulin modification and its adhesion and absorption to the intestinal tract in new born calves (39). In addition, high-yield group cows exhibited increased abundances of steroid biosynthesis, along with biosynthesis of unsaturated fatty acids, ubiquinone, and other terpenoid-quinones, although these were based on rumen metabolite profiles (40). Therefore, the microbiota functional analysis yielded results consistent with the composition analysis, both highlighting their key roles in maintaining gut health and productivity.

In the intramammary distribution of bacteria causing subclinical mastitis, CNS were the most prevalent species, consistent with previous results (15, 16). However, the CNS proportion was relatively lower (9.72–19.4% vs. 33%) (16), and the composition of specific *Staphylococcus* species differed from previous reports (15). Intramammary infection with *Staphylococcus aureus* has been identified as a risk factor for increased SCC in individual dairy cows (41) and herds (24, 42) in cases of clinical mastitis. However, SCC antigen levels on farms where *S. aureus* was detected in this study did not reveal a pattern consistent with previous studies that reported a substantial increase in SCC due to *S. aureus* infection (24, 41, 42). Thus, we suggest that the variations in the SCC levels observed in this study were mainly influenced by the differences in management practices across farms (43) rather than by *S. aureus* infection alone.

Similarity and dissimilarity analyses revealed considerable differences among farms in the structures of the fecal bacterial communities, their predicted functions, and the distribution of raw milk bacterial infections, due to management practices rather than geographic conditions and consistent with previous findings (1, 5, 6). Meanwhile recent studies on CNS have provided insights into the routes of bovine intramammary bacterial infection that the majority of fecal CNS isolates could proliferate under conditions mimicking the mammary environment (15). Furthermore, it has been suggested that fecal bacterial taxa may be heritable in a cow-to-calf manner (39) as well as within dairy herds (1). While direct evidence for a fecal-to-mammary infection route within farms is lacking, integrating similarity and dissimilarity analyses from this study with prior research on fecal bacterial heritability within farms suggests that farm-specific fecal microbiota heritability may be a key factor in the development of subclinical mastitis.

Correlation analysis showed associations among milk quality parameters, fecal bacterial composition, and predicted functions. Most core bacterial taxa exhibited negative correlations with the milk quality parameters, except for g_*UCG-010*, which showed a positive correlation with milk protein. Moreover, substantial correlations between fecal bacterial taxa and dairy cattle production profiles were observed, consistent with previous findings (4, 44). In addition, indole alkaloid biosynthesis (*ko00901*) is involved in immune regulation and promotes intestinal mucosal healing (45). Similarly, betalain biosynthesis (*ko00965*), known for its antioxidant properties, has been implicated in anti-inflammatory, anticarcinogenic, and bifidogenic effects on the gut microbiota (46). These results support the notion that core fecal bacterial taxa and their associated metabolic processes play crucial roles in microbial survival under current cattle management conditions in Korea (36, 47).

In conclusion, our findings reveal notable farm-specific differences in fecal bacterial community composition and predicted functions, as well as in the prevalence of intramammary bacterial infections, while the organic and conventional farms in the present study have maintained milk production under its respective management protocols. Although the fecal-to-mammary infection route remains a hypothesis requiring further validation, previous studies have indicated that NAS and CNS bacteria are continuously shed in dairy cow feces (16–19, 23–25, 27). Furthermore, the fecal bacterial composition and structure are closely associated with milk quality parameters, although their composition and intramammary infections appear to be highly farm-specific in the present study. Therefore, further studies are needed to elucidate their relationships by targeting both intramammary infections and environmental sources to enhance the effectiveness of mastitis prevention in dairy farming.

## Data Availability

The obtained sequencing data were deposited in the Sequence Read Archive of the National Center for Biotechnology Information and can be accessed via the SRA BioSample accession number SAMN47283394 (https://submit.ncbi.nlm.nih.gov/subs/sra/).

## Footnotes

1
http://www.dcic.co.kr

2
https://submit.ncbi.nlm.nih.gov/subs/sra/

## Supplementary material

SUPPLEMENTARY FIGURE S1 Principal component analysis (PCA) plots for the infection rates of raw milk bacteria in the organic dairy (OF) and conventional farms (CF1, CF2, and CF3). PC1 and PC2 represent principal components 1 and 2, respectively.
