# Supplementary figures and images for: Fecal microbiota profiling in organic and conventional dairy farms differing in farm-level somatic cell counts and raw milk bacterial infections
Source: Front Vet Sci. 2026 Jan 12;12:1734020. doi: 10.3389/fvets.2025.1734020 (PMC12833970; doi:10.3389/fvets.2025.1734020)

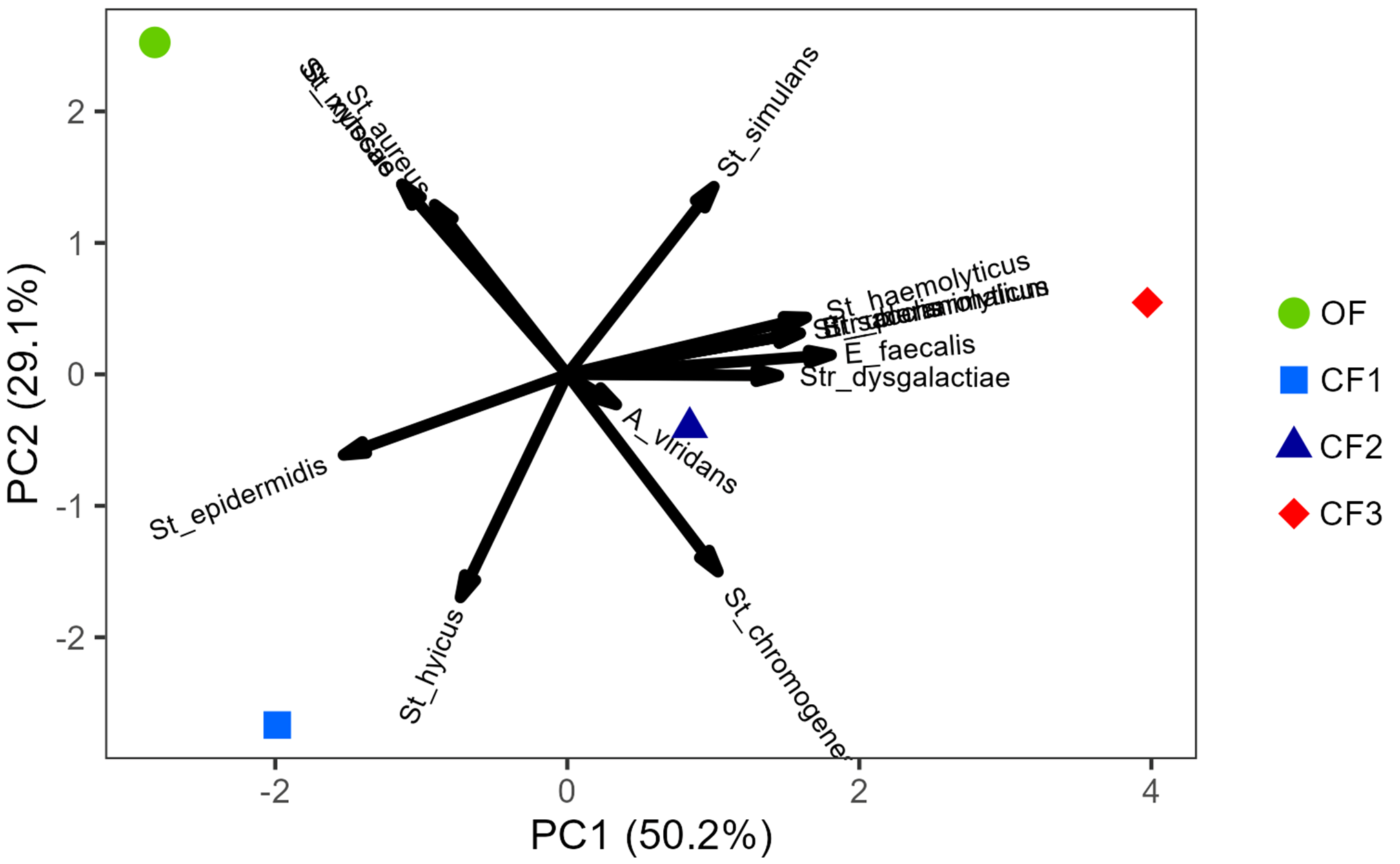

Supplement: SUPPLEMENTARY FIGURE S1 — Principal component analysis (PCA) plots for the infection rates of raw milk bacteria in the organic dairy (OF) and conventional farms (CF1, CF2, and CF3). PC1 and PC2 represent principal components 1 and 2, respectively. [file Image_1.TIF]
